# Supplementary material for: The CIP2A-TOPBP1 complex safeguards chromosomal stability during mitosis
Source: Nat Commun. 2022 Jul 16;13:4143. doi: 10.1038/s41467-022-31865-5 (PMC9288427; doi:10.1038/s41467-022-31865-5)
Supplement: Supplementary file 1 — Supplementary Information [file 41467_2022_31865_MOESM1_ESM.pdf]

Supplementary Information

The CIP2A-TOPBP1 complex safeguards chromosomal stability during mitosis

De Marco Zompit et al.

Supplementary Figures 1 – 9

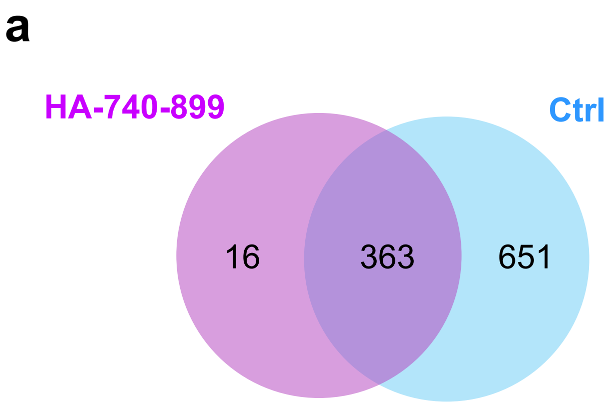

b

Accession	Protein Name
Q92547	DNA topoisomerase 2-binding protein
Q92598	Heat shock protein 105 kDa
O95757	Heat shock 70 kDa protein 4L
Q9NZL4	Hsp70-binding protein 1
O95816	BAG family molecular chaperone regulator 2
P12956	X-ray repair cross-complementing protein 6
Q96CS3	FAS-associated factor 2
Q9BSJ8	Extended synaptotagmin-1
Q15363	Transmembrane emp24 domain-containing protein 2
P28288	ATP-binding cassette sub-family D member 3
P13010	X-ray repair cross-complementing protein 5
Q8TCG1	Protein CIP2A
A0A075B6Q5	Immunoglobulin heavy variable 3
O75600	2-amino-3-ketobutyrate coenzyme A ligase, mitochondrial
P54725	UV excision repair protein RAD23 homolog A
Q86YZ3	Hornerin

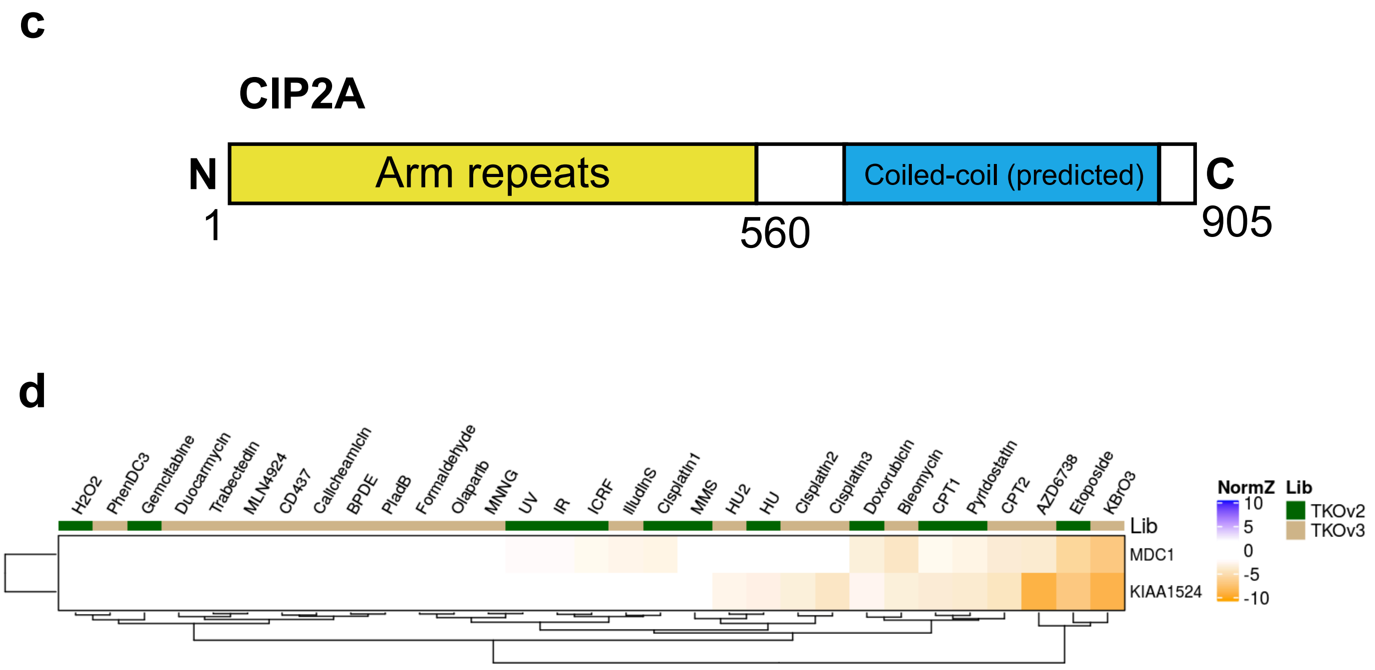

Supplementary Fig. 1 Supporting data on CIP2A is a TOPBP1 interaction partner. **a** Venn diagram of the LC-MS/MS results. **b** List of proteins identified by LC-MS/MS specifically co-immunoprecipitated by HA-TOPBP1 amino acid 740-899, but not present in the control (HA-beads alone) Bait and protein of interest are highlighted in green and yellow, respectively. **c** Schematic showing the structural domains of CIP2A. **d** Drug sensitivity correlation matrix between MDC1 and CIP2A (KIAA1524). Correlation coefficient = 0.76. Derived from https://durocher.shinyapps.io/GenotoxicScreens/ Source data are available on the ProtomeXchange Consortium via PRIDE repository with the data identifier PXD034100.

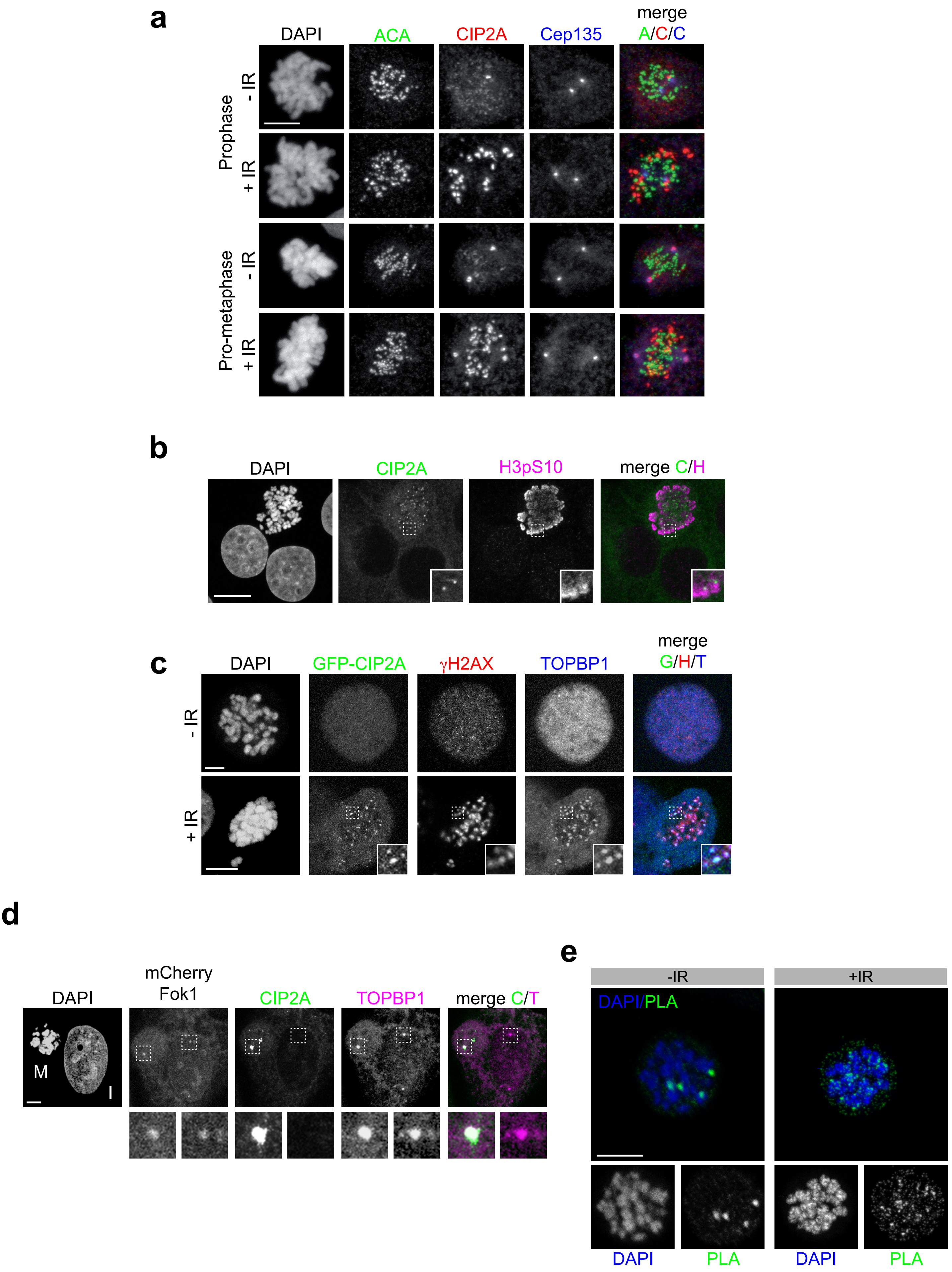
a
DAPI
ACA
CIP2A
Cep135
merge
A/C/C
Prophase
- IR
+ IR
Pro-metaphase
- IR
+ IR
b
DAPI
CIP2A
H3pS10
merge C/H
c
DAPI
GFP-CIP2A
γH2AX
TOPBP1
merge
G/H/T
- IR
+ IR
d
DAPI
mCherry
Fok1
CIP2A
TOPBP1
merge C/T
M
I
e
-IR
+IR
DAPI/PLA
DAPI
PLA
DAPI
PLA

Supplementary Fig. 2 Supporting data on CIP2A interacts with TOPBP1 at sites of DSBs in mitosis. **a** Confocal micrographs (maximum intensity projections) of DLD1 parental (WT) cells irradiated with a 2 Gy (+IR) or left untreated (-IR) and stained for CIP2A, CEP135 (to label centrosomes) or ACA (to label kinetochores). Representative cells in prophase (top) or pro-metaphase (bottom) are displayed. **b** Confocal micrographs (maximum intensity projections) of U2OS cells, irradiated with 1 Gy and stained for CIP2A and histone H3 phosphorylated on Ser 10 (H3pS10) to mark mitotic cells. **c** Confocal micrographs (maximum intensity projections) U2OS cells, transiently transfected with GFP-CIP2A, arrested in pro-metaphase by Nocodazole, irradiated with a 1 Gy (+IR) or left untreated (-IR) and stained for γH2AX (to mark DSBs) and TOPBP1. **d** Confocal micrographs (maximum intensity projections) of U2OS-DSB-reporter cells with an introduced array of the lac operator sequence and stably expressing the reporter ER-mCherry-LacI-FokI-DD, after treatment with Shield-1, 4-hydroxytamoxifen and Nocodazole for 4 hours to induce FokI clustered DSBs and stained for CIP2A and TOPBP1. The representative image contains one mitotic cell (M) and one interphase cell (I). **e** Confocal micrographs (maximum intensity projections) of U2OS cells arrested in mitosis by Nocodazole and irradiated with 1 Gy (+IR) or left untreated (-IR). Cells were subsequently processed to detect CIP2A-TOPBP1 interaction by *in situ* PLA. All scale bars = 10 μm.

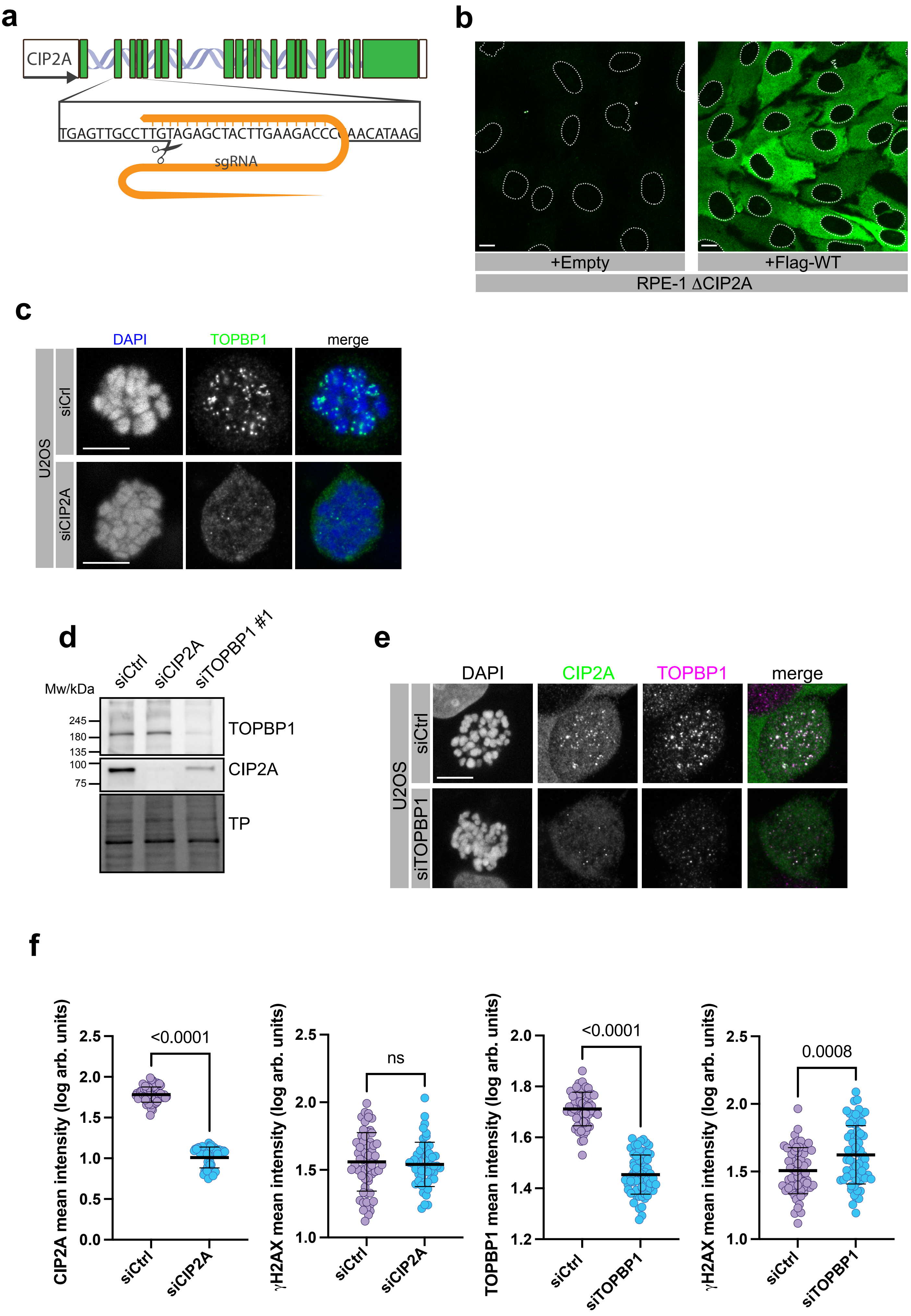
a
CIP2A
TGAGTTGCCTTGTAGAGCTACTTGAAGACCCAACATAAG
sgRNA
b
+Empty
+Flag-WT
RPE-1 ΔCIP2A
c
DAPI
TOPBP1
merge
U2OS
siCrl
siCIP2A
d
siCtrl
siCIP2A
siTOPBP1 #1
Mw/kDa
245
180
135
100
75
TOPBP1
CIP2A
TP
e
DAPI
CIP2A
TOPBP1
merge
U2OS
siCtrl
siTOPBP1
f
CIP2A mean intensity (log arb. units)
<0.0001
2.5
2.0
1.5
1.0
0.5
0.0
siCtrl
siCIP2A
γH2AX mean intensity (log arb. units)
ns
2.5
2.0
1.5
1.0
siCtrl
siCIP2A
TOPBP1 mean intensity (log arb. units)
<0.0001
2.0
1.8
1.6
1.4
1.2
siCtrl
siTOPBP1
γH2AX mean intensity (log arb. units)
0.0008
2.5
2.0
1.5
1.0
siCtrl
siTOPBP1

Supplementary Fig. 3 Supporting data on TOPBP1 accumulation at sites of mitotic DSBs is dependent on CIP2A and *vice versa*. **a** Schematic of the CIP2A knock-out strategy in RPE-1 cells. **b** Confocal micrographs of untreated RPE-1 ΔCIP2A cells, stably transduced with empty vector (+Empty) and with a lentiviral vector containing Flag-tagged CIP2A wild type cDNA (+Flag-WT) and stained for CIP2A. **c** Confocal micrographs (maximum intensity projection) of control siRNA (siCtrl) and CIP2A siRNA (siCIP2A) transfected, Nocodazole-arrested U2OS cells, 1h after irradiation with 1 Gy and stained for TOPBP1. **d** Western blots of total cell extract of U2OS cells, transfected with control siRNA (siCtrl), CIP2A siRNA (siCIP2A) and TOPBP1 siRNA (siTOPBP1 #1). **e** Confocal micrographs (maximum intensity projection) of control siRNA (siCtrl) and TOPBP1 siRNA (siTOPBP1) transfected, Nocodazole-arrested U2OS cells, 1h after irradiation with 1 Gy and stained for CIP2A and TOPBP1. **f** Quantification of γH2AX fluorescence intensities in Nocodazole-arrested U2OS cells that were transfected with control siRNA (siCtrl) and siRNAs against CIP2A (siCIP2A) and TOPBP1 (siTOPBP1 #1), 1h after irradiation with 1 Gy. Effective downregulation of CIP2A and TOPBP1 was tested by quantification of CIP2A and TOPBP1 intensities, respectively. Each datapoint corresponds to the log mean fluorescence intensity of individual cells. CIP2A depletion experiment: siCtrl n=61, siCIP2A: n=52, pooled from two independent experiments. TOPBP1 depletion experiment: siCtrl: n=62, siTOPBP: n=69, pooled from two independent experiments. Bars and error bars correspond to mean and SD. Statistical significance was assessed by two-sided unpaired t-tests ($\alpha = 0.05$). All scalebars = 10 µm. Source data are povided as a Source Data file.

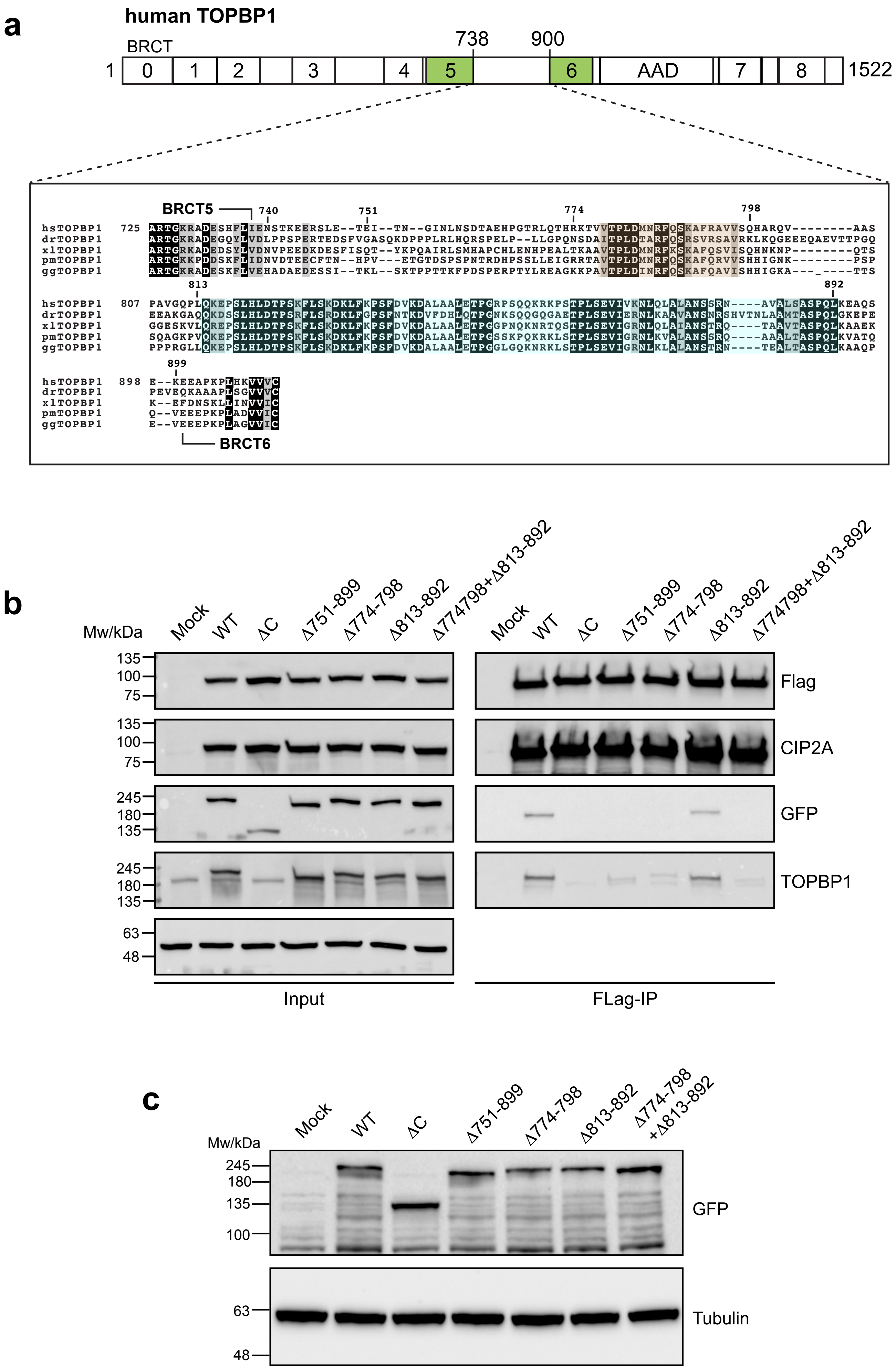
a
human TOPBP1
BRCT
1
0
1
2
3
4
5
6
AAD
7
8
1522
738
900
BRCT5
BRCT6
740
751
774
798
813
892
899
hsTOPBP1
drTOPBP1
xlTOPBP1
pmTOPBP1
ggTOPBP1
b
Mw/kDa
Mock
WT
ΔC
Δ751-899
Δ774-798
Δ813-892
Δ774798+Δ813-892
135
100
75
245
180
63
48
Flag
CIP2A
GFP
TOPBP1
Input
FLag-IP
c
Mw/kDa
Mock
WT
ΔC
Δ751-899
Δ774-798
Δ813-892
Δ774-798
+Δ813-892
245
180
135
100
63
48
GFP
Tubulin

Supplementary Fig. 4 Supporting data on two conserved sequence segments in TOPBP1 mediate its interaction with CIP2A. **a** Sequence alignment of the region between BRCT5 and BRCT6 of TOPBP1. Examples from the five vertebrate families are included (hs: *homo sapiens*, mammalia; gg: *gallus gallus*, bird; xl: *xenopus laevis*, amphibia; pm: *podarcis muralis*, reptilia; dr: *danio rerio*, fish). Highly conserved amino acids are shaded black. Two conserved regions are highlighted in color. The positions of BRCT5 and BRCT6 as well as amino acids flanking the deletion constructs are indicated. **b** Flag-immunoprecipitation from 293FT cells either Mock transfected (Mock) or co-transfected with a GFP-tagged full-length TOPBP1 wild type (WT), and various deletion mutants as indicated, and Flag-tagged CIP2A. **c** Western blots of total cell extract of U2OS cells either Mock transfected (Mock) or transfected with full-length GFP-TOPBP1 wild type (WT) and deletion mutants to probe for equal expression. Source data are povided as a Source Data file.

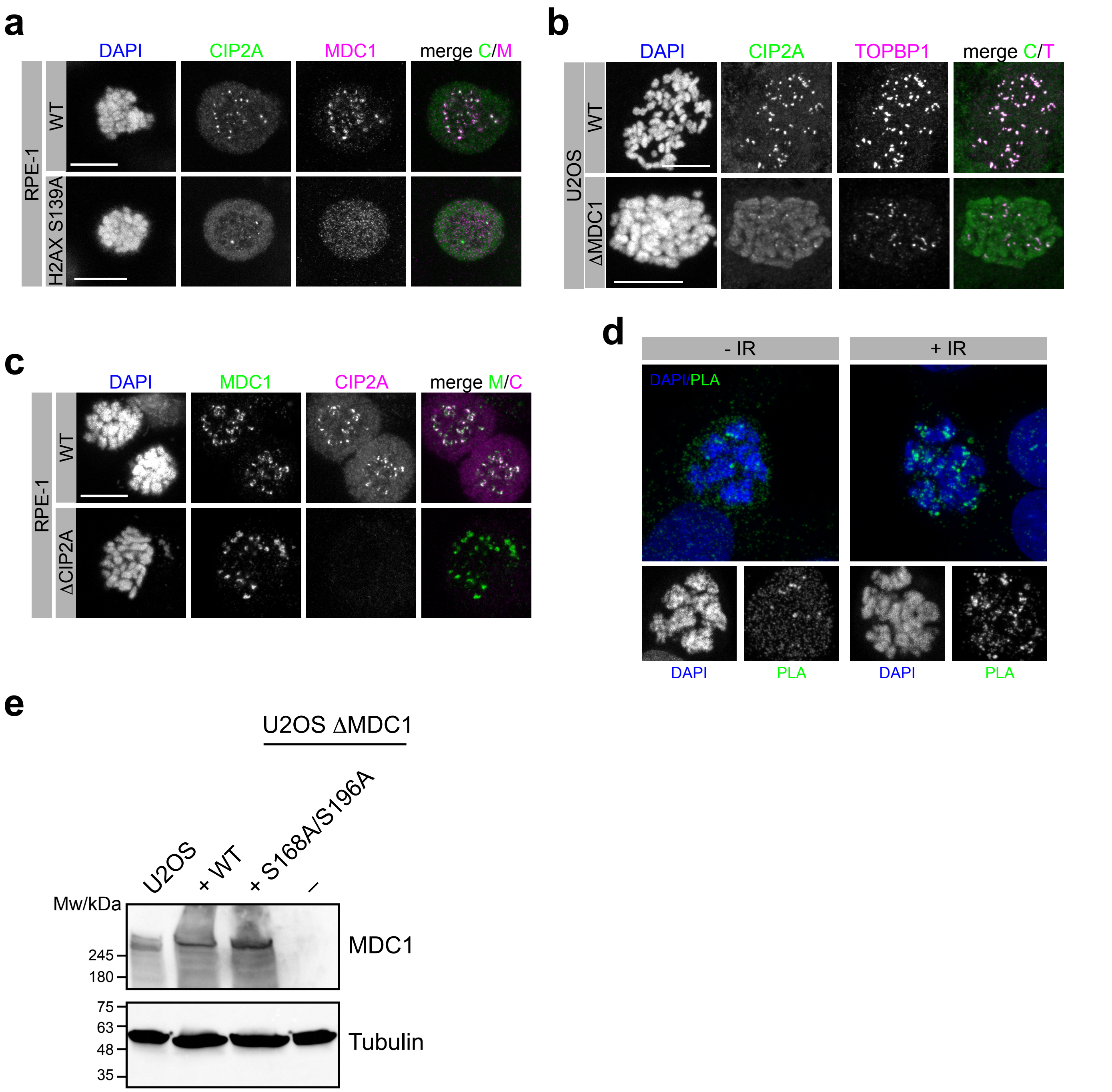

Supplementary Fig. 5 Supporting data on CIP2A-TOPBP recruitment to sites of mitotic DSBs is mediated by MDC1. **a** Confocal micrographs (maximum intensity projections) of Nocodazole-arrested RPE-1 wild type and $H2AX^{S139A/S139A}$ knock-in cells, irradiated with 1 Gy and stained for TOPBP1 and CIP2A. **b** Confocal micrographs (maximum intensity projections) of Nocodazole-arrested U2OS wild type and U2OS ΔMDC1 cells, irradiated with 1 Gy and stained for TOPBP1 and CIP2A. **c** Confocal micrographs (maximum intensity projections) of Nocodazole-arrested RPE-1 wild type and ΔCIP2A cells, treated with 1 Gy and stained for MDC1 and CIP2A. **d** Confocal micrographs (maximum intensity projections) of U2OS cells arrested in mitosis by Nocodazole and irradiated with 1 Gy (+IR) or left untreated (-IR). Cells were subsequently processed to detect CIP2A-MDC1 interaction by *in situ* PLA. **e** Western blots of total cell extract of U2OS parental cells (U2OS) and U2OS MDC1 knock-out cells cells (U2OS ΔMDC1) either not transfected (-) or stably transfected with GFP-MDC1 wild tpye (+WT) or GFP-MDC1 phosphorylation site mutant (+S168A/S196A). All scalebars = 10 µm. Source data are povided as a Source Data file.

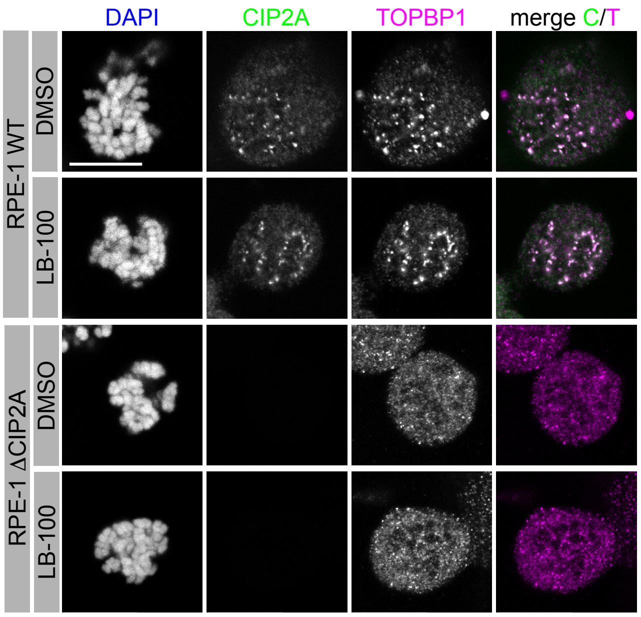

Supplementary Fig. 6 Supporting data on CIP2A controls TOPBP1 recruitment independently of PP2A. Confocal micrographs (maximum intensity projections) of Nocodazole-arrested RPE-1 wild type (RPE-1 WT) and RPE-1 CIP2A knock-out cells (RPE-1 ΔCIP2A) cells, pre-treated with the PP2A inhibitor LB-100, irradiated with 1 Gy and stained for CIP2A and TOPBP1. Scalebar = 10 µm.

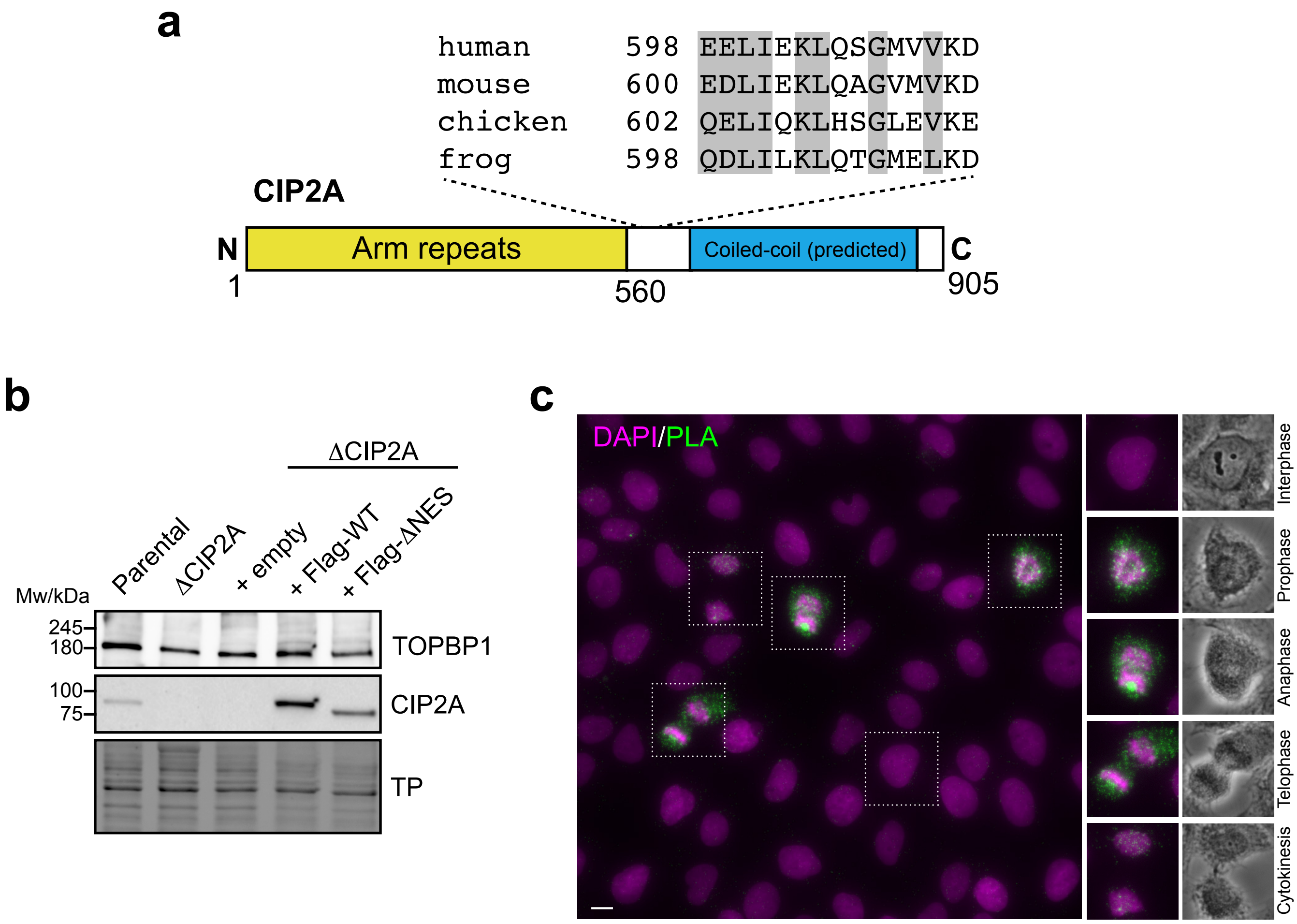

Supplementary Fig. 7 Supporting data on CRM1-dependent nuclear export sequesters CIP2A from TOPBP1 in interphase cells. **a** Schematic showing the structural domains of CIP2A and the location of a putative NES. Conserved amino acids matching the NES consensus motif are highlighted. **b** Western blots of total cell extract of parental RPE-1 cells (Parental), RPE-1 CIP2A knock-out cells (ΔCIP2A) and ΔCIP2A cells stably transduced with empty vector (+empty), Flag-tagged wild type CIP2A (Flag-WT) and Flag-tagged CIP2A lacking amino acids 561-625 (+Flag-ΔNES). TP stands for total protein on blot and served as loading control. **c** Micrograph of *in situ* PLA, using antibodies against CIP2A and TOPBP1, in unsynchronized U2OS cells. DAPI staining and phase contrast were used to visualize the cell cycle stages. All scale bars = 10 μm. Source data are povided as a Source Data file.

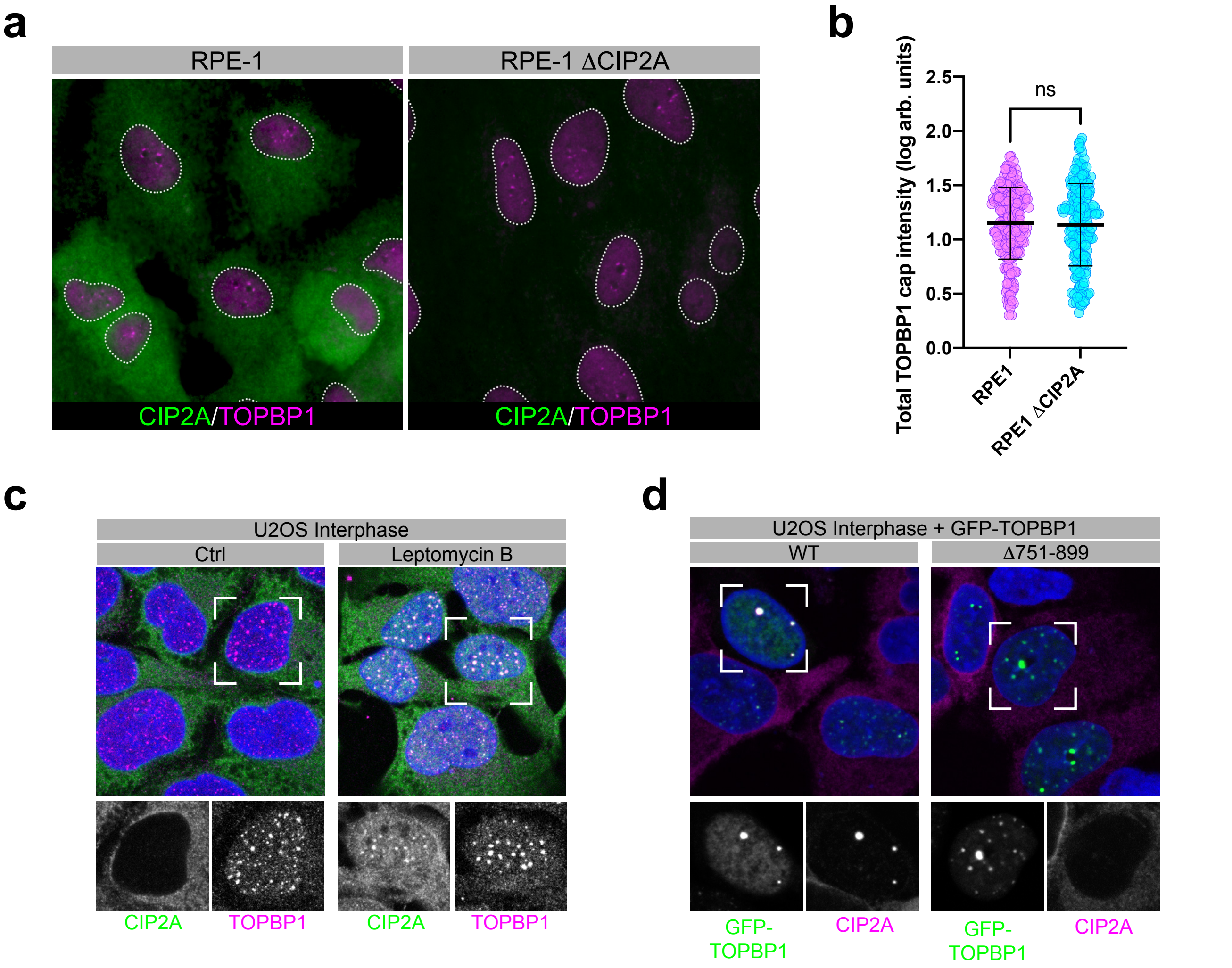

Supplementary Fig. 8 Supporting data on CIP2A is dispensable for TOPBP1 recruitment in interphase. **a** Micrographs of parental RPE-1 cells (RPE-1) and RPE-1 CIP2A knock-out cells (RPE-1 ΔCIP2A), transfected with I-Ppo1 mRNA to induce rDNA breaks and stained for CIP2A and TOPBP1. **e** Quantification of the experiment in **d**. Data points represent the log of total TOPBP1 intensity in nucleolar caps per cell (RPE-1: n=323, RPE-1 ΔCIP2A: n=329), bars and error bars represent mean and SD. Statistical significance was calculated using two-sided unpaired t-test (α=0.05); ns = not significant. **f** Confocal micrographs (maximum intensity projections) of U2OS cells pre-treated with DMSO or Leptomycin B, irradiated with 3 Gy and stained for CIP2A and TOPBP1. **g** Confocal micrographs (maximum intensity projections) of U2OS cells transfected with full-length GFP-TOPBP1 wild type (WT) and deletion mutant lacking the CIP2A interaction region (Δ751-899) and stained for CIP2A. All scale bars = 10 µm. Source data are povided as a Source Data file.

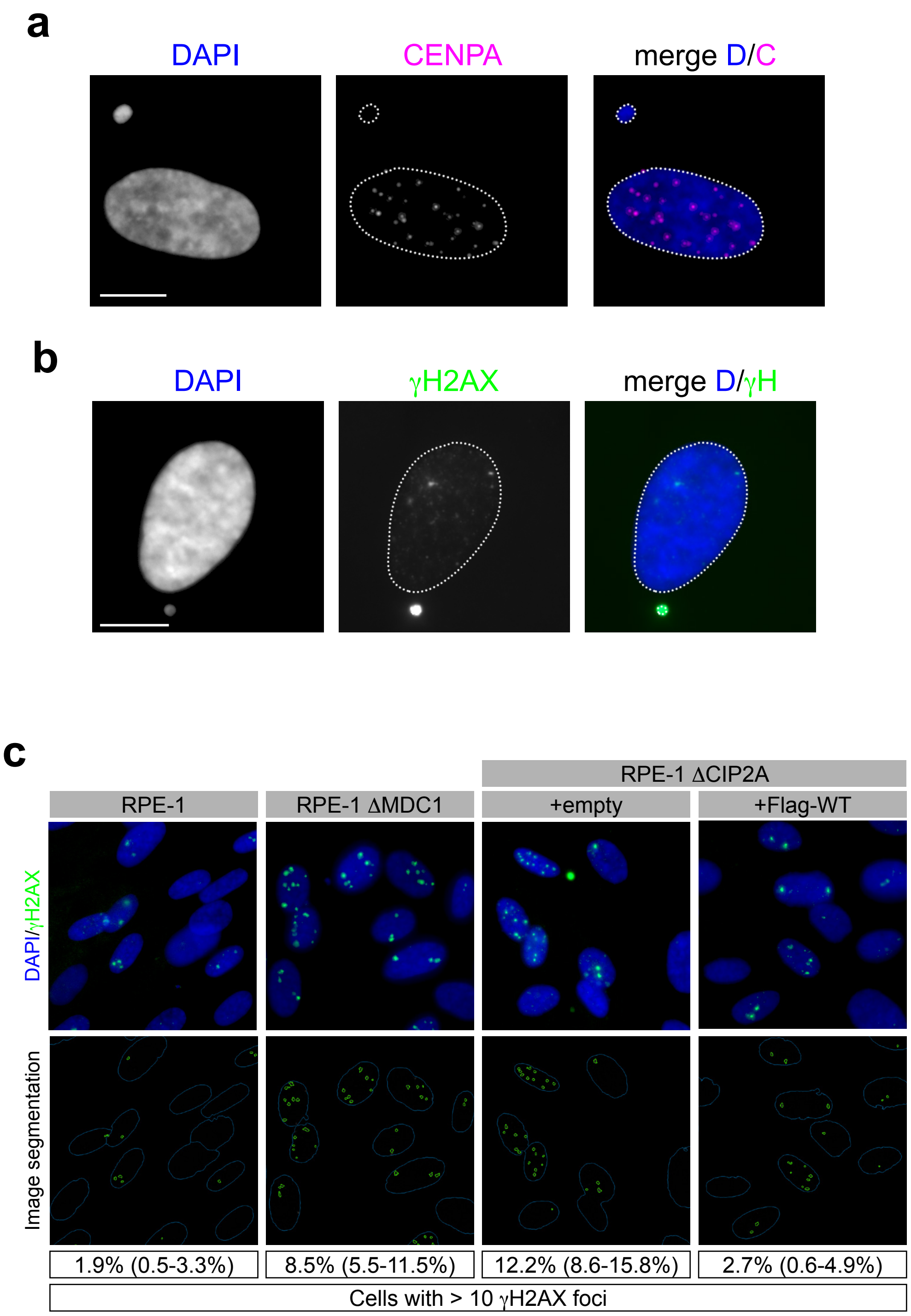

Supplementary Fig. 9 Supporting data on loss of CIP2A impairs maintenance of chromosomal stability during mitosis. **a** Confocal micrograph (maximum intensity projection) example RPE-1 ΔCIP2A cell with micronucleus, stained with CENPA antibody. **b** Confocal micrograph (maximum intensity projection) example RPE-1 ΔCIP2A cell with micro-nucleus, stained with γH2AX antibody. **c** Residual γH2AX foci 24 h after irradiation of mitotic RPE-1 parental cells (RPE-1), RPE-1 MDC1 knock-out cells (RPE-1 ΔMDC1), RPE-1 CIP2A knock-out cells (RPE-1 ΔCIP2A) cells stably transduced with empty vector (+ empty) and Flag-tagged wild type CIP2A (+ Flag-WT). Cells were arrested in G2 by RO-3306, irradiated with 0.5 Gy and released from the mitotic arrest. Upper panels: representative micrographs of cells stained for γH2AX. Lower panels: results of image segmentation for quantification. Percentage of cells with > 10 γH2AX foci are indicated, with 95% confidence intervals (RPE-1: n=368, RPE-1 ΔMDC1: n=330, + empty: n=320, + Flag-WT: n=219). All scale bars = 10 μm.
