# Supplementary material for: The CIP2A-TOPBP1 complex safeguards chromosomal stability during mitosis
Source: Nat Commun. 2022 Jul 16;13:4143. doi: 10.1038/s41467-022-31865-5 (PMC9288427; doi:10.1038/s41467-022-31865-5)
Supplement: Supplementary file 3 — Reporting Summary [file 41467_2022_31865_MOESM3_ESM.pdf]

nature research

Corresponding author(s): Manuel Stucki

Last updated by author(s): Jun 30, 2022

Reporting Summary

Nature Research wishes to improve the reproducibility of the work that we publish. This form provides structure for consistency and transparency in reporting. For further information on Nature Research policies, see our Editorial Policies and the Editorial Policy Checklist.

Statistics

For all statistical analyses, confirm that the following items are present in the figure legend, table legend, main text, or Methods section.

n/a	Confirmed	
☐	☒	The exact sample size (*n*) for each experimental group/condition, given as a discrete number and unit of measurement
☐	☒	A statement on whether measurements were taken from distinct samples or whether the same sample was measured repeatedly
☐	☒	The statistical test(s) used AND whether they are one- or two-sided<br>*Only common tests should be described solely by name; describe more complex techniques in the Methods section.*
☒	☐	A description of all covariates tested
☒	☐	A description of any assumptions or corrections, such as tests of normality and adjustment for multiple comparisons
☐	☒	A full description of the statistical parameters including central tendency (e.g. means) or other basic estimates (e.g. regression coefficient) AND variation (e.g. standard deviation) or associated estimates of uncertainty (e.g. confidence intervals)
☐	☒	For null hypothesis testing, the test statistic (e.g. *F*, *t*, *r*) with confidence intervals, effect sizes, degrees of freedom and *P* value noted<br>*Give P values as exact values whenever suitable.*
☒	☐	For Bayesian analysis, information on the choice of priors and Markov chain Monte Carlo settings
☒	☐	For hierarchical and complex designs, identification of the appropriate level for tests and full reporting of outcomes
☒	☐	Estimates of effect sizes (e.g. Cohen's *d*, Pearson's *r*), indicating how they were calculated

Our web collection on statistics for biologists contains articles on many of the points above.

Software and code

Policy information about availability of computer code

Data collection	ZEN black 2.1 (Zeiss); LAS X version 33.7.2.22383 (Leica Microsystems)
Data analysis	CellProfiler 4.0 (Broad Institute, open source); R 3.4.2 (R Development core team, open source); RStudio (RStudio PBC, 2021.09.0 Build 351); Fiji (ImageJ, open source); GraphPad Prism 9 for Mac OS X; Scaffold 4 for Mac OS X; Microsoft Excel for Mac OS X version 16.62; Adobe Photoshop CC, 23.0.0 release

For manuscripts utilizing custom algorithms or software that are central to the research but not yet described in published literature, software must be made available to editors and reviewers. We strongly encourage code deposition in a community repository (e.g. GitHub). See the Nature Research guidelines for submitting code & software for further information.

Data

Policy information about availability of data

All manuscripts must include a data availability statement. This statement should provide the following information, where applicable:
- Accession codes, unique identifiers, or web links for publicly available datasets
- A list of figures that have associated raw data
- A description of any restrictions on data availability

Numerical source data underlying Figs. 1f; 2d, f; 3c, f; 4c; 5b, d, f; 7b, d, g; 8b; 9a–e and Supplementary Figs. 3f; 8b, and uncropped scans of the gels and Western blots shown in Figs. 1c–e; 3a, d; 4a; 5c; 6a–b; 7e and Supplementary Figs. 3d; 4b–c; 5e; 7b are provided in the Source Data file linked to this paper. The proteomics data have been deposited to the ProteomeXchange Consortium via the PRIDE partner repository with the dataset identifier PXD034100 [https://www.ebi.ac.uk/pride/]. Primary imaging data have been deposited at the European Bioinformatics Institute (EBI) BioStudies database with accession number S-BIAD492 [https://www.ebi.ac.uk/biostudies/]. There are no restrictions on data availability. Source data are provided with this paper.

Field-specific reporting

Please select the one below that is the best fit for your research. If you are not sure, read the appropriate sections before making your selection.

☒ Life sciences ☐ Behavioural & social sciences ☐ Ecological, evolutionary & environmental sciences

For a reference copy of the document with all sections, see nature.com/documents/nr-reporting-summary-flat.pdf

Life sciences study design

All studies must disclose on these points even when the disclosure is negative.

Sample size	Total sample sizes required for each experiment were estimated based on the smallest differences between the means we wanted to detect, and the estimated standard deviation we expected to see. Statistical power was set to 80% and significance levels were set to 5%. The total sample sizes could then be calculated based on the table and formula provided in Motulsky H., Intuitive Biostatistics, Oxford University Press, 2014 (ISBN 978-0-19-994664-8); page 221.
Data exclusions	No data points were excluded from the analysis
Replication	All results were tested and confirmed with at least two independent experiments
Randomization	No randomization method was applied. Samples were separated in groups based on different genetic backgrounds or whether or not they were treated or not treated (small molecule inhibitors, siRNA, etc.)
Blinding	Investigators were not blinded to the group allocations because the investigators carried out the experiments.

Reporting for specific materials, systems and methods

We require information from authors about some types of materials, experimental systems and methods used in many studies. Here, indicate whether each material, system or method listed is relevant to your study. If you are not sure if a list item applies to your research, read the appropriate section before selecting a response.

Materials & experimental systems

n/a	Involved in the study	
☐	☒	Antibodies
☐	☒	Eukaryotic cell lines
☒	☐	Palaeontology and archaeology
☒	☐	Animals and other organisms
☒	☐	Human research participants
☒	☐	Clinical data
☒	☐	Dual use research of concern

Methods

n/a	Involved in the study	
☒	☐	ChIP-seq
☒	☐	Flow cytometry
☒	☐	MRI-based neuroimaging

Antibodies

Antibodies used	Primary antibodies used in this study for immunofluorescence: CIP2A (mouse, Santa Cruz, sc-80659, 1:800), γH2AX (mouse, Merck, 05-636-I, 1:500), MDC1 (rabbit, Abcam, ab11171, 1:300), TOPBP1 (rabbit, Millipore, ABE1463, 1:300), CENPA (mouse, Abcam, ab13939, 1:500), pSer10 Histone H3 (rabbit, Millipore, 06-570, 1:500) ACA (human, Antibodies Incorporated, 15-235, 1:2000), Cep135 (rabbit, home-made, 1:1000). Secondary antibodies used for immunofluorescence: Alexa Fluor 488 Goat Anti-Mouse (Life Technologies, A11029, 1:500), Alexa Fluor 568 Goat Anti-Mouse (Life Technologies A11031, 1:500), Alexa Fluor 647 Goat Anti-Mouse (Life Technologies A21235, 1:500), Alexa Fluor 488 Goat Anti-Rabbit (Life Technologies A11034, 1:500) and Alexa Fluor 568 Goat Anti-Rabbit (Life Technologies, A11036, 1:500). Primary antibodies used in this study for Western blotting: GFP (mouse, Roche, 11814460001, 1:5000), GFP (rabbit, Abcam, ab290, 1:1000), HA (rabbit, Abcam, ab9110, 1:4000), MDC1 (rabbit, Abcam, ab11171, 1:5000), MDC1 pSer168 (rabbit, custom-made, AMS Biotechnology, 1:500), MDC1 pSer196 (rabbit custom-made, 21st Century, 1:200), TOPBP1 (rabbit, Abcam, ab2402, 1:1500), CIP2A (rabbit, Cell Signaling, 14805, 1:1000), CIP2A (mouse, Santa Cruz, sc-80659, 1:1000), CRM1 (rabbit, Atlas Antibodies, HPA042933, 1:1500), Tubulin (mouse, Sigma, T6199, 1:5000), Tubulin hFAB™ rhodamine (Bio-Rad, 12004166, 1:5000), FLAG M2 (mouse, Sigma, F1804, 1:1000), pSer10 Histone H3 (rabbit, Millipore, 06-570, 1:1000), Histone H3 (rabbit, Abcam, ab1791, 1:10000). Secondary antibodies used for Western blotting: HRP-conjugated Anti-Mouse (GE Healthcare, NA934, 1:10000), HRP-conjugated Anti-Rabbit (GE Healthcare, NA934, 1:10000).
Validation	CIP2A (Santa Cruz, sc-80659): validated by the manufacturer for Western blotting. Further validated for specificity in this study by knock-out (Fig. 3a,b; Fig. 5a; Supplementary Fig. 3b).<br>TOPBP1 (Millipore, ABE1463): validated by the manufacturer for Western blotting. Further validated for specificity in Western blotting and immunofluorescence by knockdown as well as for foci formation after IR treatment and I-Ppo1 expression (Leimbacher et al. Mol Cell, 2019, 74:571-83, Mooser et al., Nat Commun 2020, 11:123). Further validated by knockdown in this study (Figs. 3d,e; 4a; Supplementary Figs. 3d,e; 4c).

H2AX pS139 (gammaH2AX; Merck, 05-636-I): validated by the manufacturer for immunofluorescence. Further validated for specificity on RPE-1 H2AX S139A knock-in cells (Leimbacher et al. Mol Cell, 2019, 74:571-83).
MDC1 (Abcam, ab11171): validated by the manufacturer for Western blotting. Further validated for specificity by knockout (Leimbacher et al. Mol Cell, 2019, 74:571-83) and in this study by knockout (Supplementary Fig. 5e).
CENPA (Abcam, ab13939): validated by the manufacturer for Western blotting. Further validated for specificity to centromeric regions in immunofluorescence on human metaphase chromosomes.
pSer10 Histone H3 (Millipore, 06-570): validated by the manufacturer for Western blotting. Further validated for mitosis specificity in Hela cells by immunofluorescence. Further validated in this study for reduction of the signal in Western blotting after IR treatment due to G2/M checkpoint activation (Fig. 1e) and for mitotic specificity in immunofluorescence (Supplementary Fig. 2b).
Cep135 (home-made): validated for centrosome localization by immunofluorescence (Bird and Hyman, J Cell Biol, 2008, 182:289-300).
GFP (Roche, 11814460001): validated by the manufacturer through comparison with a reference standard.
GFP (Abcam, ab290): validated by the manufacturer for Western blotting.
HA (Abcam, ab9110): validated by the manufacturer for Western blotting. No signal was detected in the control cells expressing no HA-tagged protein. Further validated in this study for specificity by Western blotting of cell lysates prepared from 293FT cells transfected with HA-tagged TOPBP1 fragment 740-899. No signal was present in Mock transfected cells (Fig. 1c).
MDC1 pSer168 (custom-made, AMS Biotechnology) and pSer169 (custom-made, 21st Century): validated for Western blotting (Leimbacher et al. Mol Cell, 2019, 74:571-83).
Further validated for specificity by Western blotting of cell lysates prepared from cells pre-treated with CK2 inhibitors. Further validated by Western blotting of a purified MDC1 fragment that was phosphorylated by recombinant CK2 in vitro (Leimbacher et al. Mol Cell, 2019, 74:571-83).
TOPBP1 (Abcam, ab2402): validated by the manufacturer for Western blotting. Further validated for specificity by knockdown in this study (Fig. 3d; Supplementary Fig. 3d).
CIP2A (Cell Signaling, 14805): validated by the manufacturer for Western blotting. Further validated for specificity in this study by knockout and re-expression of Flag-tagged recombinant protein (Figs. 3a; 6a and Supplementary Fig. 7b) and by knockdown (Fig. 6b).
CRM1 (Atlas Antibodies, HPA042933): validated by the manufacturer for Western blotting
Tubulin (Sigma, T6199): validated by the manufacturer for Western blotting
Tubulin hFAB™ rhodamine (Bio-Rad, 12004166): validated by the manufacturer for Western blotting using the Bio-Rad ChemiDoc MP Imaging System.
FLAG M2 (Sigma, F1804): validated by the manufacturer for Western blotting
Histone H3 (Abcam, ab1791): validated by the manufacturer for Western blotting

Eukaryotic cell lines

Policy information about cell lines

Cell line source(s)	U2OS: ATCC (gift from Dr. Steve Jackson); RPE-1 (hTERT): ATCC (gift from Dr. Alessandro Sartori); 293FT: Thermo Fisher (gift from Dr. Michael Hottiger); HeLa: ATCC; DLD1: Horizon Discovery; ExpiCHO-S: Life Technologies
Authentication	The cell lines have been authenticated based on morphological criteria.
Mycoplasma contamination	All cell lines were tested negative for mycoplasma.
Commonly misidentified lines (See ICLAC register)	No commonly misidentified cell lines were used.
